# Derivation of genetic interaction networks from quantitative phenotype data

**DOI:** 10.1186/gb-2005-6-4-r38

**Published:** 2005-03-31

**Authors:** Becky L Drees, Vesteinn Thorsson, Gregory W Carter, Alexander W Rives, Marisa Z Raymond, Iliana Avila-Campillo, Paul Shannon, Timothy Galitski

**Affiliations:** 1 Institute for Systems Biology, 1441 N. 34th Street, Seattle, WA 98103, USA

## Abstract

We have generalized the derivation of genetic-interaction networks from quantitative phenotype data. Familiar and unfamiliar modes of genetic interaction were identified and defined. A network was derived from agar-invasion phenotypes of mutant yeast. Mutations showed specific modes of genetic interaction with specific biological processes. Mutations formed cliques of significant mutual information in their large-scale patterns of genetic interaction. These local and global interaction patterns reflect the effects of gene perturbations on biological processes and pathways.

## Abstract 2

Genetic interaction networks were derived from quantitative phenotype data by analyzing agar-invasion phenotypes of mutant yeast strains, which showed specific modes of genetic interaction with specific biological processes.

## Background

Phenotypes are determined by complex interactions among gene variants and environmental factors. In biomedicine, these interacting elements take various forms: inherited and somatic human gene variants and polymorphisms, epigenetic effects on gene activity, environmental agents, and drug therapies including drug combinations. The success of predictive, preventive, and personalized medicine will require not only the ability to determine the genotypes of patients and to classify patients on the basis of molecular fingerprints of tissues. It will require an understanding of how genetic perturbations interact to affect clinical outcome. Recent advances afford the capability to perturb genes and collect phenotype data on a genomic scale [1-7]. To extract the biological information in these datasets, parallel advances must be made in concepts and computational methods to derive and analyze genetic-interaction networks. We report the development and application of such concepts and methods.

## Results and discussion

### Phenotype data and genetic interaction

A genetic interaction is the interaction of two genetic perturbations in the determination of a phenotype. Genetic interaction is observed in the relation among the phenotypes of four genotypes: a reference genotype, the 'wild type'; a perturbed genotype, *A*, with a single genetic perturbation; a perturbed genotype, *B*, with a perturbation of a different gene; and a doubly perturbed genotype, *AB*. Gene perturbations may be of any form (such as null, loss-of-function, gain-of-function, and dominant-negative). Also, two perturbations can interact in different ways for different phenotypes or under different environmental conditions.

Geneticists recognize biologically informative modes of genetic interaction, for example, epistasis and synthesis. These two modes can illustrate the general properties of genetic interactions. An epistatic interaction occurs when two single mutants have different deviant (different from wild-type) phenotypes, and the double mutant shows the phenotype of one of the single mutants. Analysis of epistatic interactions can reveal direction of information flow in molecular pathways [8]. If we represent a phenotype of a given genotype, *X*, as Φ_*X*_, then we can write a phenotype inequality representing a specific example of epistatic genetic interaction, for example, Φ_A _< Φ_*WT *_< Φ_*B *_= Φ_*AB*_. Likewise, a synthetic interaction occurs when two single mutants have a wild-type phenotype and the double mutant shows a deviant phenotype, for example, Φ_*WT *_= Φ_A _= Φ_*B *_< Φ_*AB*_. Synthetic interactions reveal mechanisms of genetic 'buffering' [1,9].

Some modes of genetic interaction are symmetric; other modes are asymmetric. This symmetry or asymmetry is evident in phenotype inequalities, and is biologically informative. Epistasis illustrates genetic-interaction asymmetry. If mutation *A *is epistatic to *B*, then *B *is hypostatic to *A*. The asymmetry of epistasis, and the form of the mutant alleles (gain or loss of function), indicates the direction of biological information flow [8]. Conversely, synthetic interactions are symmetric. If mutation *A *is synthetic with *B*, then *B *is synthetic with *A*. The symmetry of genetic synthesis reflects the mutual requirement for phenotype buffering [1,9].

The representation of genetic interactions as phenotype inequalities accommodates all possibilities without assumptions about how genetic perturbations interact. In addition, it demands quantitative (or at least ordered) phenotypes. In principle, all phenotypes are measurable; complex phenotypes (for example, different cell-type identities) are amalgamations of multiple underlying phenotypes. There is a total of 75 possible phenotype inequalities for *WT*, *A*, *B*, and *AB*. Using a hybrid approach combining the mathematical properties of phenotype inequalities and familiar genetic-interaction concepts and nomenclature, the 75 phenotype inequalities were grouped into nine exclusive modes of genetic interaction, some of which are genetically asymmetric (Additional data file 1). This approach can be extended to the interactions of more than two perturbations as well. The nine interaction modes include familiar ones: noninteractive, epistatic, synthetic, conditional, suppressive, and additive; and modes that certainly occur but, to our knowledge, have not been previously defined: asynthetic, single-nonmonotonic, and double-nonmonotonic. All interaction modes are defined in the Materials and methods; brief descriptions follow for the unfamiliar (previously undefined) modes. In asynthetic interaction, *A*, *B*, and *AB *all have the same deviant phenotype. In single-nonmonotonic interaction, a mutant gene shows opposite effects in the *WT *background and the other mutant background (for example, Φ_*WT *_< Φ_*A *_and Φ_*AB *_< Φ_*B*_). In double-nonmonotonic interaction, both mutant genes show opposite effects.

### Genetic-interaction networks

Implementation of the foregoing principles renders genetic-interaction-network derivation fully computable from data on any measured cell property with any interacting perturbations. We developed an open-source cross-platform software implementation called PhenotypeGenetics, available at [10], a plug-in for the Cytoscape general-purpose network visualization and analysis platform [11]. PhenotypeGenetics supports an XML specification for loading any dataset, allows user-defined genetic-interaction modes, and supports all of the analyses described in this paper. It was used to derive and analyze a genetic-interaction network from yeast invasion phenotype data.

In response to growth on low-ammonium agar, *Saccharomyces cerevisiae MAT***a**/*α *diploid yeast cells differentiate from the familiar ovoid single-cell growth form to a filamentous form able to invade the agar substrate [12]. Invasive filamentous-form growth is regulated by a mitogen-activated protein kinase (MAPK) kinase cascade, the Ras/cAMP pathway, and multiple other pathways [13,14]. We investigated genetic interaction among genes in these pathways and processes. Quadruplicate sets of homozygous diploid single-mutant and double-mutant yeast strains were constructed (Materials and methods). Two purposes guided the selection of genes and mutant combinations to study: to represent key pathways and processes regulating invasion; and to ensure a diversity of invasion phenotypes (non-invasive, hypo-invasive, wild type, and hyper-invasive) to permit the detection of diverse genetic interactions. A set of 19 mutant alleles of genes in key pathways controlling invasive growth, including 13 plasmid-borne dominant or multicopy wild-type alleles and 6 gene deletions, was crossed against a panel of 119 gene deletions. All mutant alleles used in this study are listed in Additional data file 2.

**Figure 1:**
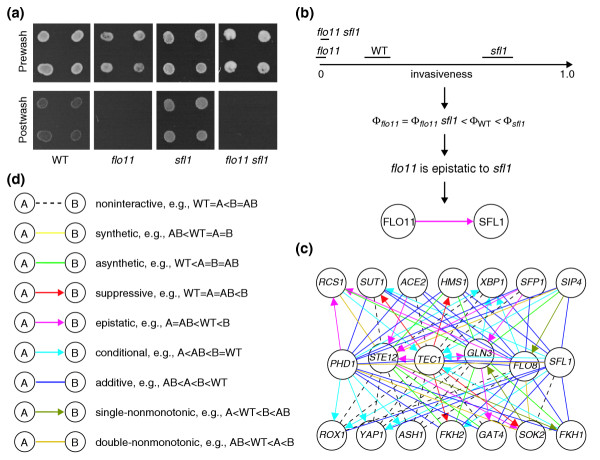
Application of the method to yeast agar invasion data to derive a genetic-interaction network. **(a) **Pre-wash and post-wash images of example genotypes in a yeast agar-invasion assay. **(b) **The invasion data shown on a phenotype axis with replicate-measurement error ranges, as a phenotype inequality, as a genetic-interaction mode, and as a graphical visualization. **(c) **Part of the network (only transcription factor genes) is shown. Nodes represent perturbed genes; edges represent genetic interactions. A key to the interactions is given in (d). **(d) **Graphical visualizations of genetic interaction modes and asymmetries, and example phenotype inequalities.

We developed a quantitative invasion-phenotype assay. Yeast agar-substrate invasion can be assessed by growing colonies on low-ammonium agar, removing cells on the agar surface by washing, and observing the remaining growth of cells inside the agar. Replicate quantitative invasion-phenotype data with error ranges were extracted from images of pre-wash and post-wash colonies. Each tested interaction was recorded as an inequality, and assigned a genetic-interaction mode. This process is detailed in the Materials and methods and illustrated in Figures 1a and 1b, using the example of the epistasis of a deletion of the *FLO11 *gene, a major determinant of invasiveness, to a deletion of the *SFL1 *gene, encoding a repressor of *FLO11*. Note that the error-bounded intervals (black bars) for the genotypes in Figure 1b are representative of the entire dataset. These errors are: *flo11*, 0.02; *flo11 sfl1*, 0.01; *WT*, 0.05; *sfl1*, 0.06. Additional data file 3 shows a plot of error values for all genotypes sorted by error magnitude. The median error is 0.04.

Graphical visualization of the genetic interactions revealed a dense complex network. For clarity, a small part of this network (interactions among transcription factors) is shown in Figure 1c, illustrating the diversity of the observed genetic interactions. Perturbed genes are nodes in the network. Each tested allele combination generates an edge representing a genetic interaction. Edge colors and arrow heads (where appropriate) indicate interaction mode and asymmetry as indicated in Figure 1d. The entire network of 127 nodes and 1,808 edges is shown in Additional data file 4. All of the underlying data, including tested interactions, genotypes, and quantitative phenotype data with error values, are listed in Additional data file 5. All nine genetic-interaction modes were observed among the 1808 interactions. Other than the noninteractive mode (with 443 occurrences), the most frequent modes were additive (347), epistatic (271), conditional (245), and suppressive (202) interaction. Lower frequencies of asynthetic (111), single-nonmonotonic (74), synthetic (62), and double-nonmonotonic (52) interaction were observed. Note that though the asynthetic, single-nonmonotonic, and double-nonmonotonic modes are not recognized by common genetic nomenclature, they occurred at substantial frequencies.

### Genetic perturbations interacting with a specific biological process

**Figure 2:**
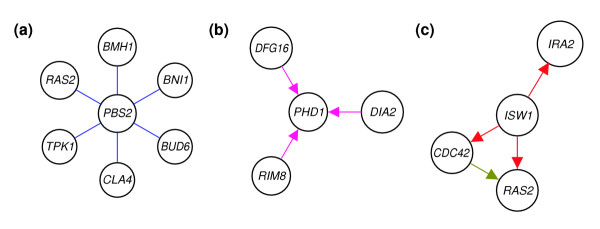
Gene perturbations show specific modes of genetic interaction with biological processes. **(a) ***PBS2 *deletion interacts additively with mutations of small-GTPase-mediated signal transduction genes. **(b) ***PHD1 *overexpression is hypostatic to deletions of invasive-growth genes. **(c) ***ISW1 *deletion suppresses the effects of perturbations of small-GTPase-mediated signal transduction genes. Key to interactions as in Figure 1d

Because genetic interactions reflect functional interactions, a genetic perturbation may interact in a specific mode with more than one gene in a specific biological process. This conjecture is supported by the finding of 'monochromatic' interaction among biological-process modules [15]. Table 1 lists 23 interactions in a specific mode between a mutant allele and a biological process. The statistical validation of these interactions is detailed in the Materials and methods. Figure 2 shows three examples. In Figure 2a, a *PBS2 *gene deletion is additive with mutations of small-GTPase-mediated signal transduction genes (*P *= 0.001). These include genes in the Rho signal transduction/cell polarity pathway (*BNI1*, *CLA4*, *BUD6*) and the Ras/cAMP signaling pathway (*RAS2, BMH1*, *TPK1*). These signaling pathways contribute to invasive growth phenotype in concert with the stress response regulated by the Pbs2 MAPK kinase [16]. In Figure 2b, deletions of invasive-growth genes *DFG16*, *RIM8*, and *DIA2 *are epistatic to overexpression of the invasion-activating Phd1 transcription factor (*P *= 0.002). The combination of this epistasis with the forms of the interacting alleles (*PHD1 *overexpression is a gain of function, whereas the others are null alleles) leads to the suggestion that *DFG16*, *RIM8*, and *DIA2 *may be regulated by Phd1. In Figure 2c, a deletion of the *ISW1 *gene suppresses the effects of perturbations of small-GTPase-mediated signal transduction genes *CDC42*, *RAS2*, and *IRA2 *(*P *= 0.005). *ISW1 *encodes an ATP-dependent chromatin-remodeling factor [17]. Halme *et al*. [18] have shown that invasiveness of yeast cells is controlled epigenetically. High-frequency spontaneous mutations of *IRA1 *and *IRA2 *relieve epigenetic silencing of invasion genes. The suppression of an *IRA2 *mutation by *ISW1 *mutation suggests the possibility that *ISW1*-dependent chromatin remodeling mediates effects of *IRA2 *mutation. Table 1 and Figure 2 illustrate local interaction patterns among mutant genes and biological processes.

### Mutually informative patterns of genetic interaction

The phenotypic consequences of combinatorial genetic perturbations are complex, in a strict sense; knowing the phenotypes of two single perturbations, there are no simple rules to know the combinatorial phenotype. Counteracting this complexity, large sets of genetic-interaction data may contain large-scale patterns. We examined the possibility that there are pairs of perturbations with mutually informative patterns of genetic interaction with their common interaction partners. In other words, knowing the interactions of one perturbation may allow one to know, to some quantifiable extent, the interactions of another perturbation, and vice versa. Mutual information, and significance thereof, was calculated for all pairs of perturbations sharing tested interactions with other genes. For all 171 pairs of the 19 mutant alleles of genes in key pathways, mutual information was based on their interactions with the panel of 119 gene deletions. Similarly, among all 7,021 pairs of the 119 gene deletions, mutual information was based on their interactions with the 19 mutant alleles of genes in key pathways. Among all possible pairs, 23 showed significant (*P *< 0.001) mutual information (Materials and methods and Additional data file 6).

**Figure 3:**
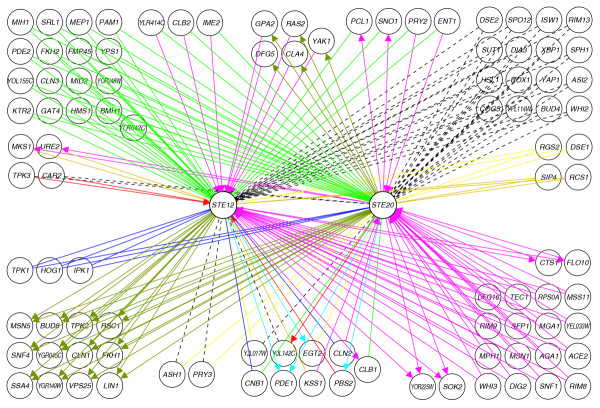
Mutually informative genes show large-scale patterns of genetic interaction. Genetic interactions of *STE12 *and *STE20 *overexpressers. Key to interactions as in Figure 1d.

The results suggest that the most mutually informative genetic-interaction patterns occur among gene perturbations with similar effects on biological processes. For example, three of the six mutant gene pairs with the most significant mutual information are overexpressers of *STE12*-*STE20*, *STE12*-*CDC42*, and *STE20*-*CDC42 *(Additional data file 6). These three genes encode central components of the MAPK signaling pathway promoting invasive filamentous-form growth [14], and they show similar patterns of genetic interaction, as exemplified by *STE12 *and *STE20 *in Figure 3. The dominant pattern is one of uniform interaction (A and B interact in the same mode with C), suggesting similar effects of the gene perturbations on the underlying molecular network. In addition, there are frequent occurrences of repeated mixed-mode interaction (A interacts in some mode with C, and B interacts in a different mode with C), suggesting that the molecular effects of gene perturbations may differ yet show consistent differences. Both uniform interaction and consistent mixed-mode interaction contribute to mutual information.

**Figure 4:**
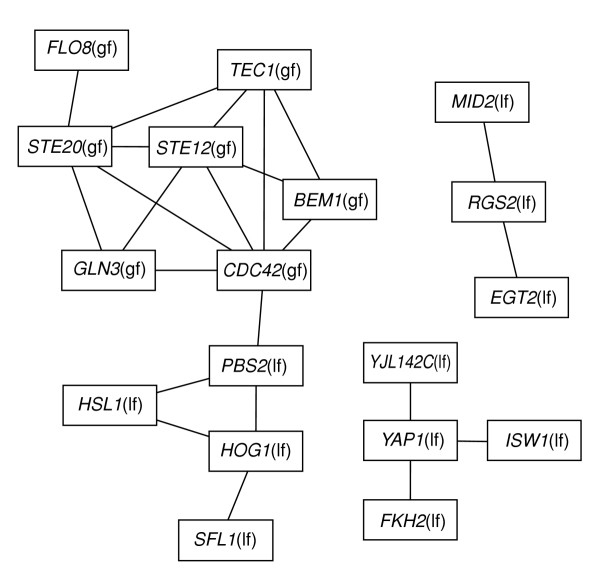
Networks of mutual information in patterns of genetic interaction show cliques. Nodes represent perturbed genes (see Additional data file 2). gf indicates a gain-of-function allele; lf indicates a loss-of-function allele. Edges connect gene pairs with significant mutual information in their patterns of genetic interaction (see Additional data file 6).

Genetic interactions are ultimately a property of a network of biological information flows. The mutual information among pathway co-member genes like *STE12 *and *STE20 *supports this. Figure 4 shows a mutual-information network of perturbed genes. Each edge indicates significant mutual information (Additional data file 6). Some of these edges connect genes in different cellular processes. For example, an edge connects the *GLN3 *gene, encoding a transcriptional regulator of nitrogen metabolism, and the *CDC42 *gene, encoding a GTPase involved in cell polarity. Such cases of mutual information suggest that in the underlying molecular network, there are important information flows between the different pathways and processes.

In addition to pairwise mutual information, there is the possibility that multiple genes may exhibit significant mutual information. The network in Figure 4 contains multiple *n*-cliques, subnetworks of *n *completely connected nodes. There is a 3-clique, including two main components (*PBS2 *and *HOG1*) of the HOG MAP-kinase pathway, and three overlapping 4-cliques (with many subcliques) containing filamentation MAPK pathway components. The *STE12*-*STE20*-*CDC42 *3-clique is in this cluster of cliques. The cliques and clusters suggest ternary and higher orders of mutual information, reflecting similarities in the global effects of perturbations on molecular information flows.

## Conclusion

The analysis of genetic interactions determining yeast invasion phenotype suggests some prospects for system-level genetics. The gene-process interactions in Table 1 and Figure 2 suggest that (as noted for epistasis and synthesis) there are characteristic network mechanisms to be found underlying familiar and unfamiliar modes of genetic interaction. Investigation of these mechanisms should provide insight on specific processes and general properties of biological networks. There are several areas for further development of the quantitative analysis of genetic interaction: first, advances in quantitative phenotype measurement and ontologies; second, reinforcement or revision of genetic-interaction mode definitions based on relevance to network mechanisms; third, extension of all genetic-interaction modes beyond phenotype ordering to incorporate parameters derived from phenotype magnitudes; and fourth, comparative genetic-interaction analyses of multiple alleles (with different effects on function) of individual genes to learn how different levels of gene activity impact the network.

The global genetic-interaction patterns illustrated in Figures 3 and 4 are readouts of the state of the underlying molecular network. Data relating genotype and phenotype are essential for understanding metabolic and information-flow paths. Genetic data, integrated with gene-activity data and molecular-interaction data, reveal direction of information flow, activations, repressions, and combinatorial controls. The genome-scale integration of molecular-wiring maps, gene-expression data, and genetic-interaction networks will enable the development of biological-network models that explicitly predict the phenotypic consequences of genetic perturbations [19].

## Materials and methods

### Strain constructions

A total of 127 genes involved in the regulation of invasion were selected for study from searches of the YPD database [20] and gene-expression profiling experiments [21,22]. 138 mutant alleles of these 127 genes, including 125 deletions and 13 plasmid-borne alleles, were assembled (Additional data file 2). Single-mutant homozygous diploid strains were constructed in the invasion-competent Σ1278b budding-yeast strain background. In quadruplicate constructions, a 19 mutant-allele subset, including the 13 plasmid-borne alleles and six of the gene deletions, was crossed against the other 119 deletions. Homozygous diploid double mutants were generated as follows.

Single-gene deletions in the invasion-competent Σ1278b yeast background were constructed. 'Barcode' gene deletion-insertion alleles [5] were PCR amplified with several hundred base pairs of flanking sequences from their noninvasive strain background. Using the G418 drug-resistance cassette of these alleles, strain G85 (*MAT***a**/*α ura3Δ0/ura3Δ0 his3Δ0::hisG/his3Δ0::hisG*) was transformed with the PCR products. Gene disruption and the presence of the KanMX4 insertion were verified by PCR. The heterozygous diploids were sporulated and the resulting tetrads were dissected and screened to select G418-resistant *MAT***a **and *MATα *haploids. These were crossed to obtain homozygous diploid gene-deletion strains.

Some of the double mutants were generated by transforming the homozygous deletion strains with either low-copy plasmids bearing dominant alleles or multicopy (2 μm-based) plasmids bearing wild-type alleles. All plasmids utilized native gene promoters. Plasmid transformations were performed using an adapted version of a multiwell transformation protocol [5,23]. Four independent transformants were stocked and assayed for each transformation. Strains were also transformed with empty vector plasmids.

The high-throughput construction of diploid homozygous double-deletion strains required the use of three drug-resistance markers to be able to select for the desired diploids and intermediate strains. For each deletion, the KanMX4 drug-resistance marker was converted to two other drug-resistance markers, HygMX4 (hygromycin resistance) and NatMX4 (nourseothricin resistance). *MATα *gene-deletion strains were transformed with the NatMX4 cassette amplified from pAG25 [24]; Nat^R ^G418^S ^transformants were stocked. *MAT***a **gene-deletion strains were transformed with the HygMX4 cassette amplified from pAG32 [24]; Hyg^R ^G418^S ^transformants were stocked.

The high-throughput construction of diploid homozygous double-deletion strains required the ability to select haploids of each mating type separately. To accomplish this, we utilized the recessive resistance to canavanine caused by the disruption of the *CAN1 *gene, encoding a transporter, in combination with fusions of the *HIS3 *ORF to the promoters of genes expressed in a specific mating type. A deletion of the *CAN1 *gene was constructed without introducing any marker genes or sequences. A double-stranded 60mer oligonucleotide containing 30 bases from the upstream region fused directly to 30 bases from the downstream region of the *CAN1 *open reading frame (5'-GTAAAAACAAAAAAAAAAAAAGGCATAGCAATATGACGTTTTATTACCTTTGATCACATT-3') was amplified with 60mer primers containing additional *CAN1 *flanking sequences (forward primer 5'-CGAAAGTTTATTTCAGAGTTCTTCA GACTTCTTAACTCCTGTAAAAACAAAAAAAAAAAA-3', reverse primer 5'-GTGTATGACTTATGAGGGTGAGAATGCGAAATGGCGTGGAAATGTGATCAAAGGTAATAA-3'). The resulting PCR product was used to transform two strains to canavanine resistance. Full deletion of the *CAN1 *gene was confirmed by PCR. This generated strains G264 (*MAT***a ***his3Δ::hisG can1Δ*) and G266 (*MATα his3Δ::hisG can1Δ*). To construct fusions of the *HIS3 *ORF to mating-type specific genes, the *S. kluyveri HIS3 *gene was amplified from pFA6-His3MX6 [25] with primers containing ORF-flanking sequences for the *MFA1 *locus (forward primer 5'-GTTTCTCGGATA AAACCAAAATAAGTACAAAGCCATCGAATAGAAATGGCAGAACCAGCCCAAAA-3', reverse primer 5'-AAGGAAGATAAAGGAGGGAGAACAACGTTTTTGTA CGCAGAAATCACATCAAAACACCTTTGTT-3') and with primers containing flanking sequences for the *MFα 1 *locus (forward primer 5'-GATTACAAACTATCAAT TTCATACACAATATAAACGATTAAAAGAATGGCAGAACCAGCCCAAAA-3', reverse primer 5'-ACAAAGTCGACTTTGTTACATCTACACTGTTGTTA TCAGTCGGGCTCACATCAAAACACCTTTGGT-3'). The resulting PCR products were used to transform G264 and G266, respectively, to create strains G544 (*MAT***a ***his3Δ::hisG can1Δmfa1::HIS3*) and G546 (*MATα his3Δ::hisG can1Δmfα1::HIS3*).

Crosses and sporulations were carried out to introduce the canavanine-resistance marker and the mating-type-specific-His^+ ^markers. *MATα *Nat^R ^deletion strains were crossed with G544. Nat^R ^Ura^+ ^diploids were selected; all were Can^S ^and His^-^. These diploids were sporulated; from random spore preparations Nat^R ^Can^R ^His^+ ^Ura^- ^*MAT***a **haploids were identified. *MAT***a **Hyg^R ^deletion strains were crossed with G546. These diploids were sporulated; from random spore preparations Hyg^R ^Can^R ^His^+ ^Ura^- ^*MATα *haploids were identified.

To make the diploid homozygous double-deletion strains, a series of high-throughput crosses and sporulations, in which all the desired intermediate cell types and deletion genotypes could be selected, was carried out [1]. At all stages, multiple strains were individually verified. Nat^R ^*MAT***a **single-deletion strains were crossed to an array of *MATα *G418^R ^single-deletion strains. Hyg^R ^*MATα *single-deletion strains were crossed to an array of *MAT***a **G418^R ^single-deletion strains. From these crosses Nat^R ^G418^R ^diploids and Hyg^R ^G418^R ^diploids were selected, respectively. Diploids from each cross were sporulated. Haploid double-deletion strains were selected: His^+ ^Can^R ^(*MAT***a **haploid) G418^R ^Nat^R ^double-deletion segregants, and His^+ ^Can^R ^(*MATα *haploid) G418^R ^Hyg^R ^double-deletion segregants, respectively. The resulting arrays of *MAT***a **and *MATα *haploid double-deletion strains were mated and subjected to selection for G418^R^, Nat^R^, and Hyg^R ^to generate diploid homozygous double-deletion strains.

### Assay of yeast invasiveness

Strains were inoculated from frozen stocks into liquid media in 96-well plates and incubated 18 hours at 30°C. Each plate included at least eight wells containing wild-type controls. Cells were transferred with a 96-Floating-Pin Replicator and colony copier (V & P Scientific) onto SLAD agar [12] in an omnitray. Each 96-well plate was pinned in quadruplicate, resulting in a total of 384 colonies per SLAD-agar plate. Note that each genotype was constructed in quadruplicate and assayed on separate plates. Therefore, each genotype was assayed with a total of 16 replicates. Plates were incubated for 4 days at 30°C. After incubation, cell material was removed from the agar surface while rinsing the plate under running water. A 300 d.p.i. grayscale image of each plate was generated before and after the wash by placing the plate face down on a flatbed scanner and scanning with transmitted light. Images were inverted using Adobe Photoshop 6 and saved as TIFF files for quantitative image analysis.

### Processing of invasion-assay data

Colony growth and invasion were quantified using Dapple [26], software originally designed for the analysis of DNA microarray images. Each post-wash image was analyzed simultaneously with the corresponding pre-wash image to enable reliable definition of colony boundaries and direct comparison of cell material. Subtraction of local background intensity yielded un-normalized values for growth (*G*) and invasion (*U*) and invasiveness ratio (*R *= *U*/*G*). A normalization factor for each plate, *N*_*p*_, was obtained from multiple wild-type controls on each plate. For each replicate *q *on plate *p*, we obtained the wild-type invasiveness ratio *R*^*wt*^_*q*,*p *_= *U*^*wt*^_*q*,*p*_/*G*^*wt*^_*q*,*p *_and defined the plate normalization factor *N*_*p *_as *N*_*p *_= median_*q*_(R^*wt*^_*q*,*p*_)/median_*p*_(median_*q*_(R^*wt*^_*q*,*p*_)). For a given genotype *g*, the normalized invasiveness ratio is given by *R*^*g*^_*q*,*p *_= (*U*^*g*^_*q*,*p*_/*G*^*g*^_*q*,*p*_)/*N*_*p *_= *R*^*g*^_*i *_where in the final equality we renumbered into a single ordinal index the *N *replicates *i *= 1,2,..,*N *(*N *≤ 16). We excluded any genotype *g *for which *N *< 5 due, for example, to deficient growth. From normalized data, we derived phenotype values and measurement errors. We obtained the median ratio *R*^*g *^= median_*i*_(*R*^*g*^_*i*_), and the median absolute deviation MAD^*g *^= MAD(*R*^*g*^_*i*_) = median_*i*_(|*R*^*g*^_*i*_-*R*^*g*^|). As a lower bound in error estimates we used MAD^*Q *= 0.1^, the tenth percentile of all MAD^*g*^. Thus, the phenotype values are reported as *R*^*g*^, with error *E*^*g *^= max(MAD^*g*^, MAD^*Q *= 0.1^). The frequencies of genetic-interaction modes were insensitive to increases of the error lower bound to the 50th percentile. Directed checks of individual components of the automated processing were made throughout. These included: visual inspection of each individual image, check of colony morphology, spot checking of well-characterized individual strains from start to finish in the analysis pipeline, screening for systematic errors in assay intensities. We confirmed that the plate-wise normalization did not lead to error amplification due to division by small numbers.

### Derivation of phenotype inequalities

The following steps were carried out using PhenotypeGenetics software. Phenotypes and errors of genotypes *WT*, *A*, *B*, and *AB *[(*R*^*wt*^,*E*^*wt*^), (*R*^*A*^,*E*^*A*^), (*R*^*B*^,*E*^*B*^), and (*R*^*AB*^, *E*^*AB*^)] were assigned a phenotype inequality relation. This was done by first defining the error-bounded interval *I*^*g *^= [*R*^*g*^-*E*^*g*^, *R*^*g*^+*E*^*g*^] for each genotype. All pairs of genotypes were assigned an equality, Φ^*g*1 ^= Φ^*g*2 ^if interval *I*^*g*1 ^overlapped with *I*^*g*2^. Transitivity of equalities (if a = b and b = c, then a = c) was applied to yield disjoint groups of phenotype equalities. Inequalities, greater than (>) or less than (<) were assigned for the relations between equality groups. The resulting inequalities were assigned to genetic-interaction modes and asymmetries as described below. The results of all tests of genetic interaction were rendered as a graph as illustrated in Figure 1d. The entire resulting network is shown in Additional data file 4. One can obtain PhenotypeGenetics software or use it to analyze the invasion network at [10].

### Modes of genetic-interaction

The 75 possible phenotype inequalities were assigned to modes of genetic interaction based on computable criteria. For each mode, we list the criterion for the inclusion of a phenotype inequality. In these criteria, 'background' refers to a genotype with its complement of wild-type and mutant genes, into which other genetic perturbations are added, and 'effect' refers to a change in a phenotype, either an increase or a decrease, upon a single genetic perturbation of a background. In the examples below, additional cases may be generated by operations such as exchanging *A *and *B*, or reversing the effect of both alleles (for example reversing the effect of the *A *mutant gene with Φ_*WT*_< Φ_*A *_gives Φ_*A *_< Φ_*WT*_). Additional data file 1 lists each of the 75 phenotype inequalities and their assigned genetic-interaction mode and asymmetries. Figure 1d shows graph visualizations for all nine genetic-interaction modes.

#### Noninteractive interaction

*A *has no effect in the *WT *and *B *backgrounds (for example, Φ_*WT *_= Φ_*A *_< Φ_*B *_= Φ_*AB*_), or *B *has no effect in the *A *and *WT *backgrounds, or both hold true (5 inequalities).

#### Epistatic interaction

*A *and *B *have different effects (in terms of direction or magnitude) on the wild-type background and the double mutant has the same phenotype as either *A *or *B *(for example, Φ_*A *_< Φ_*WT *_< Φ_*B *_= Φ_*AB*_) (12 inequalities).

#### Conditional interaction

*A *has an effect only in the *B *background, or the *B *mutant has an effect only in the *A *background (12 inequalities).

#### Suppressive interaction

*A *has an effect on *WT*, but that effect is abolished by adding the suppressor *B*, which itself shows no single-mutant effect (for example, Φ_*WT *_= Φ_*B *_= Φ_*AB *_*<*Φ_*A*_); or, the corresponding holds under exchange of *A *and *B *(4 inequalities).

#### Additive interaction

Single-mutant effects combine to give a double-mutant effect as per Φ_*WT *_<Φ_*A*_= Φ_*B*_<Φ_*AB*_, Φ_*B *_< Φ_*WT *_= Φ_*AB *_< Φ_*A*_, Φ_*WT *_< Φ_*A *_< Φ_*B *_< Φ_*AB*_, Φ_*B *_< Φ_*WT *_< Φ_*AB *_< Φ_*A*_, and all additional inequalities obtained by interchanging *A *and *B*, or reversing the effect of both *A *and *B *(12 inequalities).

#### Synthetic interaction

*A *and *B *have no effect on the *WT *background, but the *AB *combination has an effect (2 inequalities).

#### Asynthetic interaction

*A*, *B*, and the *AB *combination all have the same effect on the *WT *background (2 inequalities).

#### Single-nonmonotonic interaction

*B *shows opposing effects in the *WT *and *A *backgrounds (for example, Φ_*B *_> Φ_*WT *_and Φ_*AB*_< Φ_*A*_); or, *A *shows opposing effects in the *WT *and *B *backgrounds, but not both (8 inequalities).

#### Double-nonmonotonic interaction

Both *A *and *B *show opposing effects in the *WT *background and the background with the other mutant gene (18 inequalities).

### Genetic interaction with biological processes

To identify statistically significant correlations between a given allele's interaction modes and biological processes, the neighbors of every allele in the network were queried for interaction class and Gene Ontology (GO) Consortium database annotations [27]. Each interaction class is defined by the interaction mode and direction, if any. For example, 'A suppresses B' and 'A is suppressed by B' are placed in different interaction classes. There are 13 interaction classes and 9 interaction modes (described above). Likelihood values were computed to find over-represented class-annotation pairings within each set of nearest neighbors, and *P*-values were assigned relative to a cumulative hypergeometric distribution. The result was a computer-generated list of biological statements relating genes, interaction classes, and target annotations, with entries such as 'A loss-of-function mutation of *HSL1 *is suppressed by mutations of cell wall organization and biogenesis genes (-log_10_*P *= 2.52).' These are listed in tabular form in Table 1.

To calibrate the significance of the results, a parallel calculation was performed for every test in the network in which the fractional probabilities of each possible outcome were added to an overall distribution of *P*-values for the entire network. For example, if a given mutation interacts with *N *others, *N*_*C *_of the interactions being of class *C *and *N*_*A *_of those neighbors carrying annotation *A*, there is a finite set of outcomes for *N*_*CA*_, the number of neighbor mutations with annotation *A *connected via interaction *C*. The possible values of *N*_*CA *_follow a discrete hypergeometric distribution, and summing these distributions over all tests in the network yields a formally randomized distribution of P-values which has been constrained by the topology of the actual network. The distributions, real and theoretical, of -log_10_*P *values were then compared by performing a chi-square test between comparable histograms. These tests showed a strong excess for -log_10_*P *> 1.8.

### Mutual information of genetic interaction patterns

We calculated the mutual information [28] of pairs of genetic perturbations. Each perturbation, *X*, has an observed discrete probability distribution of interaction classes (defined by mode and direction) with its tested interaction partners, *P*(*x*), where *x *∈ *X*, the set of interaction classes of perturbation *X*, and:



Mutual information, *I*, of a pair of perturbations, *A *and *B*, is the relative entropy of their joint probability distribution relative to their product probability distribution. Thus:



Significance of mutual information was tested independently for each allele pair by computing the likelihood of obtaining the observed score in randomly permuted data. To remove bias due to our selection of mutant alleles, randomized data were constrained by keeping the wild type and two single-mutant phenotypes fixed and replacing interaction classes only with classes that are consistent with the observed single-mutant phenotypes. The choice among possible replacement classes was weighted by observed frequency in the entire network. Empirical tests showed randomized mutual information scores to be normally distributed, and multiple randomizations were carried out to determine a mean and standard deviation to characterize the distribution for each tested allele pair. *P*-values were then calculated as the probability of finding a mutual information score at or above the observed score. Allele pairs with probabilities below the cutoff of *P *< 0.001 are listed in Additional data file 6, and shown as a graph in Figure 4.

## Additional data files

The following data are available with the online version of this paper. Additional data file 1 is a table showing 75 genetic-interaction inequalities in nine modes of genetic interaction. Additional data file 2 lists the gene perturbations used in this study. Additional data file 3 is a figure plotting phenotype error values in the entire dataset. Additional data file 4 shows the entire genetic interaction network derived from yeast invasion-phenotype data. Additional data file 5 lists phenotype data for all tested interactions. Additional data file 6 lists mutual information in genetic-interaction patterns.

## Supplementary Material

Additional File 1 A table showing 75 genetic-interaction inequalities in nine modes of genetic interaction. As described in Materials and methods, all 75 possible phenotype inequalities were classified into nine modes of genetic interaction. The results are listed here. Click here for file

Additional File 2 Gene perturbations used in this study. This file lists all genes, mutant alleles, and allele forms (for example, null, gain-of-function, etc.) Click here for file

Additional File 3 Phenotype error values in the entire dataset. This plot shows the phenotype error values (Materials and methods) plotted against percentile of all genotypes ordered by error magnitude. Click here for file

Additional File 4 Entire genetic interaction network derived from yeast invasion-phenotype data. Figure 1c shows a small part of the genetic-interaction network. This file contains an image including all tested interactions. Click here for file

Additional File 5 Phenotype data for all tested interactions. This file lists all tested genetic interactions as well as the phenotype and error values for all genotypes, *WT*, *A*, *B*, and *AB*. Click here for file

Additional File 6 Mutual information in genetic-interaction patterns. This file lists the mutual information, and significance, among pairs of genes connected by edges in Figure 4. Click here for file

## Figures and Tables

**Table 1:** Genetic interactions of mutant genes with biological processes

Gene	Form*	Interaction	Biological process	-log_10_*P*
*PBS2*	null	Additive	Signal transduction	2.99
*PBS2*	null	Additive	Small gtpase mediated signal transduction	2.96
*STE12*	gf	Single-nonmonotonic to	Protein targeting	2.87
*STE11*	da	Noninteractive	Cell cycle	2.73
*PHD1*	gf	Hypostatic to	Invasive growth	2.68
*PDE2*	null	Noninteractive	Protein amino acid phosphorylation	2.56
*HSL1*	null	Suppressed by	Cell wall organization and biogenesis	2.52
*STE20*	gf	Single-nonmonotonic to	Protein targeting	2.31
*EGT2*	null	Conditioned by	Invasive growth	2.30
*ISW1*	null	Suppresses	Small gtpase mediated signal transduction	2.30
*CLB1*	null	Noninteractive	Protein metabolism	2.30
*STE11*	da	Suppresses	Cell surface receptor linked signal transduction	2.28
*BEM1*	gf	Conditioned by	Nucleobase, nucleoside, nucleotide and nucleic acid metabolism	2.25
*PBS2*	null	Additive	Ras protein signal transduction	2.24
*PBS2*	null	Additive	Sporulation	2.24
*TEC1*	gf	Synthetic	Intracellular signaling cascade	2.19
*IPK1*	null	Additive	M phase	1.95
*TEC1*	null	Epistatic to	Metabolism	1.94
*TEC1*	gf	Conditioned by	Carbohydrate metabolism	1.94
*TEC1*	gf	Conditioned by	Ras protein signal transduction	1.94
*BUD4*	null	Noninteractive	Establishment of cell polarity	1.94
*HMS1*	null	Noninteractive	Protein amino acid phosphorylation	1.83
*YGR045C*	null	Noninteractive	Protein amino acid phosphorylation	1.83

*gf, gain-of-function; da, dominant-active.

## References

[B1] Tong AH, Evangelista M, Parsons AB, Xu H, Bader GD, Page N, Robinson M, Raghibizadeh S, Hogue CW, Bussey H (2001). Systematic genetic analysis with ordered arrays of yeast deletion mutants.. Science.

[B2] Tong AH, Lesage G, Bader GD, Ding H, Xu H, Xin X, Young J, Berriz GF, Brost RL, Chang M (2004). Global mapping of the yeast genetic interaction network.. Science.

[B3] Fraser AG, Kamath RS, Zipperlen P, Martinez-Campos M, Sohrmann M, Ahringer J (2000). Functional genomic analysis of *C. elegans *chromosome I by systematic RNA interference.. Nature.

[B4] Tewari M, Hu PJ, Ahn JS, Ayivi-Guedehoussou N, Vidalain PO, Li S, Milstein S, Armstrong CM, Boxem M, Butler MD (2004). Systematic interactome mapping and genetic perturbation analysis of a C. elegans TGF-beta signaling network.. Mol Cell.

[B5] Winzeler EA, Shoemaker DD, Astromoff A, Liang H, Anderson K, Andre B, Bangham R, Benito R, Boeke JD, Bussey H (1999). Functional characterization of the S. cerevisiae genome by gene deletion and parallel analysis.. Science.

[B6] Strausberg RL, Schreiber SL (2003). From knowing to controlling: a path from genomics to drugs using small molecule probes.. Science.

[B7] Swedlow JR, Goldberg I, Brauner E, Sorger PK (2003). Informatics and quantitative analysis in biological imaging.. Science.

[B8] Avery L, Wasserman S (1992). Ordering gene function: the interpretation of epistasis in regulatory hierarchies.. Trends Genet.

[B9] Hartman JLt, Garvik B, Hartwell L (2001). Principles for the buffering of genetic variation.. Science.

[B10] Galitski Lab. http://labs.systemsbiology.net/galitski.

[B11] Shannon P, Markiel A, Ozier O, Baliga NS, Wang JT, Ramage D, Amin N, Schwikowski B, Ideker T (2003). Cytoscape: a software environment for integrated models of biomolecular interaction networks.. Genome Res.

[B12] Gimeno CJ, Ljungdahl PO, Styles CA, Fink GR (1992). Unipolar cell divisions in the yeast *S. cerevisiae *lead to filamentous growth: regulation by starvation and RAS.. Cell.

[B13] Lengeler KB, Davidson RC, D'Souza C, Harashima T, Shen WC, Wang P, Pan X, Waugh M, Heitman J (2000). Signal transduction cascades regulating fungal development and virulence.. Microbiol Mol Biol Rev.

[B14] Gancedo JM (2001). Control of pseudohyphae formation in *Saccharomyces cerevisiae*.. FEMS Microbiol Rev.

[B15] Segre D, DeLuna A, Church GM, Kishony R (2005). 'Monochromatic' modularity of epistatic interaction network in yeast metabolism.. Nat Genet.

[B16] O'Rourke SM, Herskowitz I (1998). The Hog1 MAPK prevents cross talk between the HOG and pheromone response MAPK pathways in *Saccharomyces cerevisiae*.. Genes Dev.

[B17] Tsukiyama T, Palmer J, Landel CC, Shiloach J, Wu C (1999). Characterization of the imitation switch subfamily of ATP-dependent chromatin-remodeling factors in *Saccharomyces cerevisiae*.. Genes Dev.

[B18] Halme A, Bumgarner S, Styles C, Fink GR (2004). Genetic and epigenetic regulation of the FLO gene family generates cell-surface variation in yeast.. Cell.

[B19] Galitski T (2004). Molecular networks in model systems.. Annu Rev Genomics Hum Genet.

[B20] Csank C, Costanzo MC, Hirschman J, Hodges P, Kranz JE, Mangan M, O'Neill K, Robertson LS, Skrzypek MS, Brooks J (2002). Three yeast proteome databases: YPD, PombePD, and CalPD (MycoPathPD).. Methods Enzymol.

[B21] Galitski T, Saldanha AJ, Styles CA, Lander ES, Fink GR (1999). Ploidy regulation of gene expression.. Science.

[B22] Prinz S, Avila-Campillo I, Aldridge C, Srinivasan A, Dimitrov K, Siegel AF, Galitski T (2004). Control of yeast filamentous-form growth by modules in an integrated molecular network.. Genome Res.

[B23] *Saccharomyces *Genome Deletion Project. http://www-sequence.stanford.edu/group/yeast_deletion_project/transprot.html.

[B24] Goldstein AL, McCusker JH (1999). Three new dominant drug resistance cassettes for gene disruption in *Saccharomyces cerevisiae*.. Yeast.

[B25] Wach A (1996). PCR-synthesis of marker cassettes with long flanking homology regions for gene disruptions in *S. cerevisiae*.. Yeast.

[B26] Dapple: image analysis software for DNA microarrays. http://www.cs.wustl.edu/~jbuhler/research/dapple.

[B27] Ashburner M, Ball CA, Blake JA, Botstein D, Butler H, Cherry JM, Davis AP, Dolinski K, Dwight SS, Eppig JT (2000). Gene ontology: tool for the unification of biology. The Gene Ontology Consortium.. Nat Genet.

[B28] Shannon CE (1948). A mathematical theory of communication.. Bell System Tech J.

