# Supplementary material for: Derivation of genetic interaction networks from quantitative phenotype data
Source: Genome Biol. 2005 Mar 31;6(4):R38. doi: 10.1186/gb-2005-6-4-r38 (PMC1088966; doi:10.1186/gb-2005-6-4-r38)
Supplement: Additional File 1 — A table showing 75 genetic-interaction inequalities in nine modes of genetic interaction. As described in Materials and methods, all 75 possible phenotype inequalities were classified into nine modes of genetic interaction. The results are listed here. [file gb-2005-6-4-r38-S1.pdf]

Additional data file 1. 75 genetic-interaction inequalities in 9 modes of genetic interaction.

Inequality	Mode	Asymmetry[a]
A=AB<WT=B	noninteractive	
B=AB<WT=A	noninteractive	
WT=A<B=AB	noninteractive	
WT=B<A=AB	noninteractive	
WT=A=B=AB	noninteractive	
AB<WT=A=B	synthetic	
WT=A=B<AB	synthetic	
A=B=AB<WT	asynthetic	
WT<A=B=AB	asynthetic	
A<WT=B=AB	suppressive	A ← B
B<WT=A=AB	suppressive	A → B
WT=A=AB<B	suppressive	A → B
WT=B=AB<A	suppressive	A ← B
A<B=AB<WT	epistatic	A ← B
B<A=AB<WT	epistatic	A → B
A=AB<B<WT	epistatic	A → B
B=AB<A<WT	epistatic	A ← B
A<WT<B=AB	epistatic	A ← B
B<WT<A=AB	epistatic	A → B
A=AB<WT<B	epistatic	A → B
B=AB<WT<A	epistatic	A ← B
WT<A<B=AB	epistatic	A ← B
WT<B<A=AB	epistatic	A → B
WT<A=AB<B	epistatic	A → B
WT<B=AB<A	epistatic	A ← B
WT=A<AB<B	conditional	A ← B
WT=B<AB<A	conditional	A → B
WT=A<B<AB	conditional	A ← B
WT=B<A<AB	conditional	A → B
A<WT=B<AB	conditional	A → B
B<WT=A<AB	conditional	A ← B
AB<WT=A<B	conditional	A ← B
AB<WT=B<A	conditional	A → B
A<AB<WT=B	conditional	A → B
B<AB<WT=A	conditional	A ← B
AB<A<WT=B	conditional	A → B
AB<B<WT=A	conditional	A ← B
A<AB<WT<B	additive	
B<AB<WT<A	additive	
A<WT<AB<B	additive	
B<WT<AB<A	additive	

A<WT=AB<B	additive	
B<WT=AB<A	additive	
AB<A<B<WT	additive	
AB<B<A<WT	additive	
WT<A<B<AB	additive	
WT<B<A<AB	additive	
AB<A=B<WT	additive	
WT<A=B<AB	additive	
A<AB<B<WT	single-nonmonotonic	A ← B
B<AB<A<WT	single-nonmonotonic	A → B
A<WT<B<AB	single-nonmonotonic	A → B
B<WT<A<AB	single-nonmonotonic	A ← B
AB<A<WT<B	single-nonmonotonic	A ← B
AB<B<WT<A	single-nonmonotonic	A → B
WT<A<AB<B	single-nonmonotonic	A → B
WT<B<AB<A	single-nonmonotonic	A ← B
A<B<AB<WT	double-nonmonotonic	
B<A<AB<WT	double-nonmonotonic	
A<B<WT<AB	double-nonmonotonic	
B<A<WT<AB	double-nonmonotonic	
A<B<WT=AB	double-nonmonotonic	
B<A<WT=AB	double-nonmonotonic	
A=B<AB<WT	double-nonmonotonic	
A=B<WT<AB	double-nonmonotonic	
A=B<WT=AB	double-nonmonotonic	
AB<WT<A<B	double-nonmonotonic	
AB<WT<B<A	double-nonmonotonic	
AB<WT<A=B	double-nonmonotonic	
WT<AB<A<B	double-nonmonotonic	
WT<AB<B<A	double-nonmonotonic	
WT<AB<A=B	double-nonmonotonic	
WT=AB<A<B	double-nonmonotonic	
WT=AB<B<A	double-nonmonotonic	
WT=AB<A=B	double-nonmonotonic	

[a] Blank table fields indicate symmetric interactions. Arrows indicate a conventional representation of asymmetry. These conventions are used in visual representations throughout this work.
