# Supplementary material for: Derivation of genetic interaction networks from quantitative phenotype data
Source: Genome Biol. 2005 Mar 31;6(4):R38. doi: 10.1186/gb-2005-6-4-r38 (PMC1088966; doi:10.1186/gb-2005-6-4-r38)
Supplement: Additional File 2 — Gene perturbations used in this study. This file lists all genes, mutant alleles, and allele forms (for example, null, gain-of-function, etc.) [file gb-2005-6-4-r38-S2.pdf]

Additional data file 2. Gene perturbations used in this study.

Allele[a]	Gene	Locus	Allele Form
pbs2::bcKanMX4	*PBS2*	*YJL128C*	null
sfl1::bcKanMX4	*SFL1*	*YOR140W*	null
hsl1::bcKanMX4	*HSL1*	*YKL101W*	null
ipk1::bcKanMX4	*IPK1*	*YDR315C*	null
ira2::bcKanMX4	*IRA2*	*YOL081W*	null
hog1::bcKanMX4	*HOG1*	*YLR113W*	null
RAS2A22	*RAS2*	*YNL098C*	dominant-negative
RSR1N16	*RSR1*	*YGR152C*	dominant-negative
KSS1K42R	*KSS1*	*YGR040W*	dominant-negative
STE11-4	*STE11*	*YLR362W*	dominant-active
TEC1$^+$mc	*TEC1*	*YBR083W*	gain-of-function
CDC42$^+$mc	*CDC42*	*YLR229C*	gain-of-function
FLO8$^+$mc	*FLO8*	*YER109C*	gain-of-function
STE20$^+$mc	*STE20*	*YHL007C*	gain-of-function
STE12$^+$mc	*STE12*	*YHR084W*	gain-of-function
PHD1$^+$mc	*PHD1*	*YKL043W*	gain-of-function
MSN1$^+$mc	*MSN1*	*YOL116W*	gain-of-function
GLN3$^+$mc	*GLN3*	*YER040W*	gain-of-function
BEM1$^+$mc	*BEM1*	*YBR200W*	gain-of-function
dig2::bcKanMX4	*DIG2*	*YDR480W*	null
tec1::bcKanMX4	*TEC1*	*YBR083W*	null
flo1::bcKanMX4	*FLO1*	*YAR050W*	null
clb1::bcKanMX4	*CLB1*	*YGR108W*	null
yer124c::bcKanMX4	*DSE1*	*YER124C*	null
gpr1::bcKanMX4	*GPR1*	*YDL035C*	null
pcl1::bcKanMX4	*PCL1*	*YNL289W*	null
tpk1::bcKanMX4	*TPK1*	*YJL164C*	null
cln1::bcKanMX4	*CLN1*	*YMR199W*	null
fkh1::bcKanMX4	*FKH1*	*YIL131C*	null
cts1::bcKanMX4	*CTS1*	*YLR286C*	null
dbr1::bcKanMX4	*DBR1*	*YKL149C*	null
fkh2::bcKanMX4	*FKH2*	*YNL068C*	null
mep2::bcKanMX4	*MEP2*	*YNL142W*	null
flo11::bcKanMX4	*MUC1*	*YIR019C*	null
rgs2::bcKanMX4	*RGS2*	*YOR107W*	null
ash1::bcKanMX4	*ASH1*	*YKL185W*	null
egt2::bcKanMX4	*EGT2*	*YNL327W*	null
ras2::bcKanMX4	*RAS2*	*YNL098C*	null
pde1::bcKanMX4	*PDE1*	*YGL248W*	null
sok2::bcKanMX4	*SOK2*	*YMR016C*	null
flo10::bcKanMX4	*FLO10*	*YKR102W*	null
ure2::bcKanMX4	*URE2*	*YNL229C*	null
msn1::bcKanMX4	*MSN1*	*YOL116W*	null

sip4::bcKanMX4	*SIP4*	*YJL089W*	null
ipk1::bcKanMX4	*IPK1*	*YDR315C*	null
pry2::bcKanMX4	*PRY2*	*YKR013W*	null
sno1::bcKanMX4	*SNO1*	*YMR095C*	null
gpa2::bcKanMX4	*GPA2*	*YER020W*	null
pry3::bcKanMX4	*PRY3*	*YJL078C*	null
cla4::bcKanMX4	*CLA4*	*YNL298W*	null
rcs1::bcKanMX4	*RCS1*	*YGL071W*	null
rox1::bcKanMX4	*ROX1*	*YPR065W*	null
dfg16::bcKanMX4	*DFG16*	*YOR030W*	null
aga1::bcKanMX4	*AGA1*	*YNR044W*	null
msn5::bcKanMX4	*MSN5*	*YDR335W*	null
mss11::bcKanMX4	*MSS11*	*YMR164C*	null
cnb1::bcKanMX4	*CNB1*	*YKL190W*	null
pde2::bcKanMX4	*PDE2*	*YOR360C*	null
ent1::bcKanMX4	*ENT1*	*YDL161W*	null
isw1::bcKanMX4	*ISW1*	*YBR245C*	null
hms1::bcKanMX4	*HMS1*	*YOR032C*	null
gat4::bcKanMX4	*GAT4*	*YIR013C*	null
bmh1::bcKanMX4	*BMH1*	*YER177W*	null
rim9::bcKanMX4	*RIM9*	*YMR063W*	null
dia3::bcKanMX4	*DIA3*	*YDL024C*	null
bud6::bcKanMX4	*BUD6*	*YLR319C*	null
bni1::bcKanMX4	*BNI1*	*YNL271C*	null
rsc1::bcKanMX4	*RSC1*	*YGR056W*	null
mep3::bcKanMX4	*MEP3*	*YPR138C*	null
mep1::bcKanMX4	*MEP1*	*YGR121C*	null
dia1::bcKanMX4	*DIA1*	*YMR316W*	null
pgu1::bcKanMX4	*PGU1*	*YJR153W*	null
cna1::bcKanMX4	*CNA1*	*YLR433C*	null
bud8::bcKanMX4	*BUD8*	*YLR353W*	null
ypl114w::bcKanMX4	*YPL114W*	*YPL114W*	null
ktr2::bcKanMX4	*KTR2*	*YKR061W*	null
tpk2::bcKanMX4	*TPK2*	*YPL203W*	null
ylr042c::bcKanMX4	*YLR042C*	*YLR042C*	null
yor225w::bcKanMX4	*YOR225W*	*YOR225W*	null
yel033w::bcKanMX4	*YEL033W*	*YEL033W*	null
spo12::bcKanMX4	*SPO12*	*YHR152W*	null
yps1::bcKanMX4	*YPS1*	*YLR120C*	null
ynl051w::bcKanMX4	*COG5*	*YNL051W*	null
rim8::bcKanMX4	*RIM8*	*YGL045W*	null
ygr149w::bcKanMX4	*YGR149W*	*YGR149W*	null
yap1::bcKanMX4	*YAP1*	*YML007W*	null
ylr414c::bcKanMX4	*YLR414C*	*YLR414C*	null
ssa4::bcKanMX4	*SSA4*	*YER103W*	null
pam1::bcKanMX4	*PAM1*	*YDR251W*	null

whi3::bcKanMX4	*WHI3*	*YNL197C*	null
ygr045c::bcKanMX4	*YGR045C*	*YGR045C*	null
rps0A::bcKanMX4	*RPS0A*	*YGR214W*	null
whi2::bcKanMX4	*WHI2*	*YOR043W*	null
sut1::bcKanMX4	*SUT1*	*YGL162W*	null
car2::bcKanMX4	*CAR2*	*YLR438W*	null
sph1::bcKanMX4	*SPH1*	*YLR313C*	null
yhr143w::bcKanMX4	*DSE2*	*YHR143W*	null
ynl159c::bcKanMX4	*ASI2*	*YNL159C*	null
bud4::bcKanMX4	*BUD4*	*YJR092W*	null
dfg5::bcKanMX4	*DFG5*	*YMR238W*	null
ime2::bcKanMX4	*IME2*	*YJL106W*	null
yol155c::bcKanMX4	*YOL155C*	*YOL155C*	null
kss1::bcKanMX4	*KSS1*	*YGR040W*	null
mid2::bcKanMX4	*MID2*	*YLR332W*	null
mga1::bcKanMX4	*MGA1*	*YGR249W*	null
ydl222c::bcKanMX4	*FMP45*	*YDL222C*	null
tos11::bcKanMX4	*YOR248W*	*YOR248W*	null
srl1::bcKanMX4	*SRL1*	*YOR247W*	null
yhr156c::bcKanMX4	*LIN1*	*YHR156C*	null
yjl017w::bcKanMX4	*YJL017W*	*YJL017W*	null
xbp1::bcKanMX4	*XBP1*	*YIL101C*	null
yak1::bcKanMX4	*YAK1*	*YJL141C*	null
tpk3::bcKanMX4	*TPK3*	*YKL166C*	null
cln2::bcKanMX4	*CLN2*	*YPL256C*	null
pbs2::bcKanMX4	*PBS2*	*YJL128C*	null
ace2::bcKanMX4	*ACE2*	*YLR131C*	null
clb2::bcKanMX4	*CLB2*	*YPR119W*	null
mrp21::bcKanMX4	*MRP21*	*YBL090W*	null
rim13::bcKanMX4	*RIM13*	*YMR154C*	null
yjl142c::bcKanMX4	*YJL142C*	*YJL142C*	null
mks1::bcKanMX4	*MKS1*	*YNL076W*	null
mih1::bcKanMX4	*MIH1*	*YMR036C*	null
sfp1::bcKanMX4	*SFP1*	*YLR403W*	null
hmi1::bcKanMX4	*HMI1*	*YOL095C*	null
msm1::bcKanMX4	*MSM1*	*YGR171C*	null
hog1::bcKanMX4	*HOG1*	*YLR113W*	null
akr1::bcKanMX4	*AKR1*	*YDR264C*	null
gln3::bcKanMX4	*GLN3*	*YER040W*	null
hsl1::bcKanMX4	*HSL1*	*YKL101W*	null
snf1::bcKanMX4	*SNF1*	*YDR477W*	null
elm1::bcKanMX4	*ELM1*	*YKL048C*	null
sfl1::bcKanMX4	*SFL1*	*YOR140W*	null
yir002c::bcKanMX4	*MPH1*	*YIR002C*	null
snf4::bcKanMX4	*SNF4*	*YGL115W*	null
cln3::bcKanMX4	*CLN3*	*YAL040C*	null

ira2::bcKanMX4	*IRA2*	*YOL081W*	null
vps25::bcKanMX4	*VPS25*	*YJR102C*	null
dia2::bcKanMX4	*DIA2*	*YOR080W*	null

[a] The first 19 mutant alleles were crossed against the remaining 119 deletion alleles. *bcKanMX4* indicates a “barcode” deletion-insertion allele. *mc* indicates the allele is borne on a multicopy plasmid.
