# Supplementary material for: Derivation of genetic interaction networks from quantitative phenotype data
Source: Genome Biol. 2005 Mar 31;6(4):R38. doi: 10.1186/gb-2005-6-4-r38 (PMC1088966; doi:10.1186/gb-2005-6-4-r38)
Supplement: Additional File 3 — Phenotype error values in the entire dataset. This plot shows the phenotype error values (Materials and methods) plotted against percentile of all genotypes ordered by error magnitude. [file gb-2005-6-4-r38-S3.pdf]

**Additional data file 3**

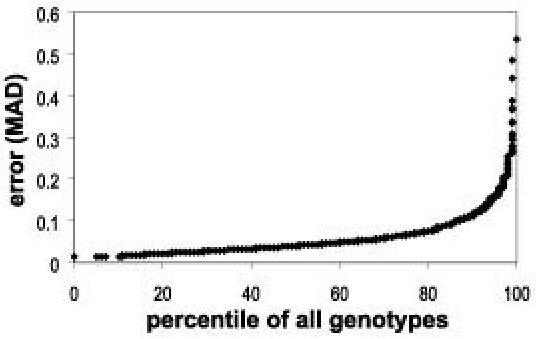


**Phenotype error values in the entire data set.** This plot shows the phenotype error values (median absolute deviation; see Materials and Methods) plotted against percentile of all genotypes ordered by error magnitude.
