# Supplementary material for: Derivation of genetic interaction networks from quantitative phenotype data
Source: Genome Biol. 2005 Mar 31;6(4):R38. doi: 10.1186/gb-2005-6-4-r38 (PMC1088966; doi:10.1186/gb-2005-6-4-r38)
Supplement: Additional File 4 — Entire genetic interaction network derived from yeast invasion-phenotype data. Figure 1c shows a small part of the genetic-interaction network. This file contains an image including all tested interactions. [file gb-2005-6-4-r38-S4.pdf]

Additional data file 4

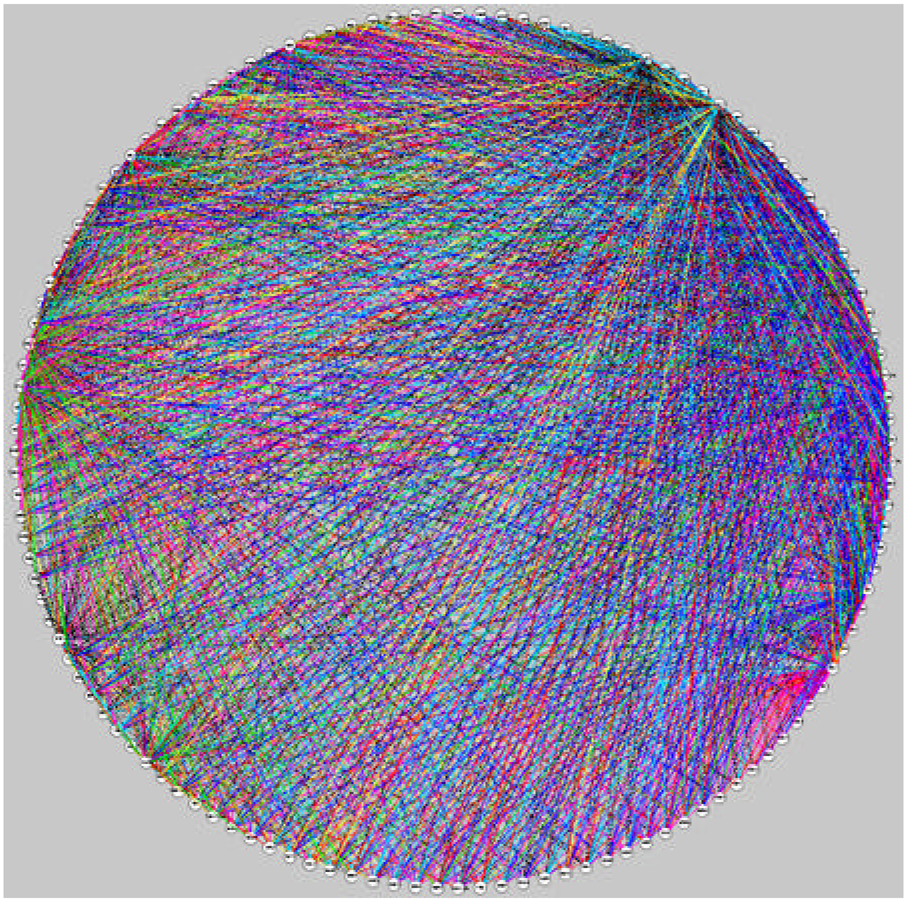

Genetic interaction network derived from yeast invasion-phenotype data.

Genetic interactions are represented as in Figure 1D. The entire genetic network is shown. The network can be explored and analyzed interactively at http://labs.systemsbiology.net/galitski.
