# Supplementary material for: Derivation of genetic interaction networks from quantitative phenotype data
Source: Genome Biol. 2005 Mar 31;6(4):R38. doi: 10.1186/gb-2005-6-4-r38 (PMC1088966; doi:10.1186/gb-2005-6-4-r38)
Supplement: Additional File 6 — Mutual information in genetic-interaction patterns. This file lists the mutual information, and significance, among pairs of genes connected by edges in Figure 4. [file gb-2005-6-4-r38-S6.pdf]

Additional data file 6. Mutual information in genetic-interaction patterns.

Gene1[a]	**Gene2**[a]	**Common**[b]	**Mutual Info.**[c]	**$-\log_{10}P$**
STE20(gf)	*STE12(gf)*	99	1.8	16.3
PBS2(lf)	*HOG1(lf)*	101	1.2	14.1
CDC42(gf)	*BEM1(gf)*	99	1.0	9.5
STE20(gf)	*CDC42(gf)*	100	1.5	9.2
PBS2(lf)	*HSL1(lf)*	95	1.5	8.9
STE12(gf)	*CDC42(gf)*	101	1.5	8.0
FLO8(gf)	*STE20(gf)*	100	1.3	6.7
STE20(gf)	*TEC1(gf)*	99	0.9	6.6
STE12(gf)	*GLN3(gf)*	99	1.4	6.3
TEC1(gf)	*BEM1(gf)*	95	0.7	5.0
SFL1(lf)	*HOG1(lf)*	75	0.8	4.8
STE12(gf)	*BEM1(gf)*	97	0.8	4.4
CDC42(gf)	*GLN3(gf)*	101	1.3	4.3
HOG1(lf)	*HSL1(lf)*	99	0.9	4.3
CDC42(gf)	*PBS2(lf)*	86	1.0	3.5
FKH2(lf)	*YAP1(lf)*	18	2.2	3.5
TEC1(gf)	*CDC42(gf)*	99	0.8	3.3
ISW1(lf)	*YAP1(lf)*	17	2.4	3.3
RGS2(lf)	*MID2(lf)*	15	2.3	3.3
STE20(gf)	*GLN3(gf)*	98	1.2	3.3
YJL142C(lf)	*YAP1(lf)*	17	2.1	3.2
EGT2(lf)	*RGS2(lf)*	16	2.1	3.1
STE12(gf)	*TEC1(gf)*	98	0.8	3.0

[a] "gf" indicates a gain-of-function allele. "lf" indicates a loss-of-function allele.
[b] The number of interaction partners tested in common.
[c] Units are bits.
